# Swelling and Erythema of the Lower Extremities

**DOI:** 10.6004/jadpro.2016.7.1.10

**Published:** 2016-01-01

**Authors:** Beth Eaby-Sandy

**Affiliations:** Abramson Cancer Center, Hospital of the University of Pennsylvania, Philadelphia, Pennsylvania

## History 

Mr. T is a 78-year-old male who initially presented with stage IV oligometastatic non–small cell lung cancer (NSCLC) with adrenal metastasis. He is a retired pharmacist who comes to appointments with his wife. His medical history includes hypertension, emphysema, esophageal reflux, and hyperlipidemia. He initially presented with abdominal pain. Upon workup, a CT image of the chest and abdomen revealed a solitary adrenal metastasis and one lung lesion. He had laparoscopic resection of the adrenal metastasis. However, shortly afterward, he was found to have metastatic disease in the surgical bed and progression of the lung mass.

Mr. T was started on pemetrexed (Alimta) and carboplatin for six cycles, followed by maintenance pemetrexed. He then developed brain metastases and underwent gamma knife treatment to three brain lesions. Subsequently, he was started on gemcitabine and carboplatin for progression of disease in the chest. After four cycles, he went on to receive maintenance gemcitabine.

## Chief Complaint 

After 2 months of maintenance gemcitabine chemotherapy, Mr. T began to complain of lower extremity swelling, erythema, and minor discomfort. He denied difficulty walking and did not have a fever. After treatment with oral cephalexin, the lower extremity edema improved, and he remained on chemotherapy. However, 4 weeks later, the swelling and erythema in his lower extremities recurred (see photo above).

Treatment with further cephalexin did not improve his symptoms, and he was switched to oral clindamycin. Four days after initiation of clindamycin, his symptoms continued to worsen, manifesting as increased discomfort, increased edema, and now difficulty walking and inability to wear shoes.

**Figure 1:**
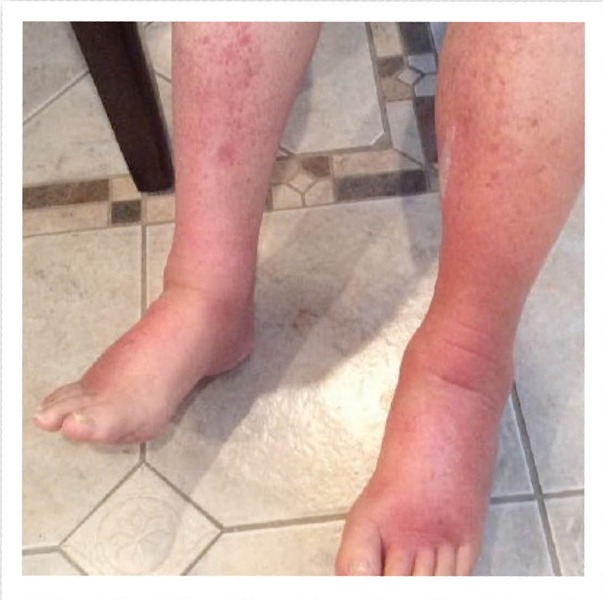
Swelling and Erythema of the lower extremities

##  Review of Systems and Physical Exam

While Mr. T has a history of hypertension, there is no history of other cardiac disease or congestive heart failure. Nonpitting edema, progressive from +1 to now +3, and erythema were present in both of Mr. T’s legs, with the left greater than the right. The edema did not improve with elevation of his legs, and his feet did not fit into his shoes. He described the pain as minor, about 3 out of 10, though at this point he was experiencing some difficulty walking.

Mr. T was afebrile, with a normal white blood cell count. Respiratory, cardiac, and neurologic exams were within normal limits. Doppler ultrasound of the lower extremities was performed and found to be negative for thrombosis. Mr. T was then admitted to the hospital for intravenous antibiotics and continued workup.

**Figure 2:**
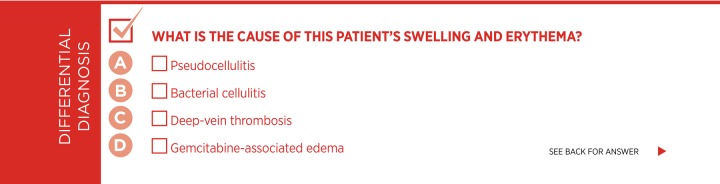
What is the cause of this patient’s swelling and erythema?

## Correct Answer: A 

**Pseudocellulitis.** Pseudocellulitis is simply a noninfectious cellulitis. Gemcitabine has been known to cause a rash in 30% of patients, yet less than 1% are grade 3 (Lilly, 2014). Rashes seen with gemcitabine chemotherapy have been described as dermatitis, often from radiation recall, to myositis or erysipeloid reactions (Tan, Bunce, Liles, & Gold, 2007). Radiation recall dermatitis reactions occur on areas of the body that were radiated in the past. However, there are case reports in the literature of lower extremity pseudocellulitis in an area of the body that had never been radiated (Singh & Hampole, 2012; Korniyenko, Lozada, Ranade, & Sandhu, 2012; Obeid & Venugopal, 2013). One school of thought is that pseudocellulitis could occur in lower extremities due to drug accumulation in the subcutaneous tissues, especially where there may be impaired lymphatic drainage (Korniyenko et al., 2012).

##  Explanation of Incorrect Answers

**Bacterial cellulitis.** Though Mr. T responded to the first round of oral antibiotics, his symptoms persisted and did not respond to a second round of oral antibiotics. There was no elevated white blood cell count and no fever. Though this is a more likely diagnosis in most patients, the clinical picture here suggests further workup. It is important to keep this high on the differential diagnosis, though, considering the fact that this is an immunosuppressed population and that the consequences of ignoring a bacterial infection could lead to sepsis.

**Deep-vein thrombosis.** While the lower-extremity swelling coupled with a diagnosis of lung cancer raises significant concern for deep-vein thrombosis and should absolutely be ruled out, Mr. T had negative ultrasounds of the lower extremities.

**Gemcitabine-associated edema.** In an analysis of five clinical trials evaluating gemcitabine as a single agent, peripheral edema was reported in 20% of patients, though less than 1% of patients discontinued the drug due to edema (Lilly, 2014). Given the erythema, difficulty ambulating, and the lack of improvement in swelling upon elevation of the lower extremities, gemcitabine-associated edema is likely not the main cause of Mr. T’s symptoms.

Other oncology drugs that could cause lower-extremity edema or skin breakdown of the lower extremities in a patient with NSCLC include pemetrexed, docetaxel, and some of the targeted therapies as well (Lacouture, 2012).

## Management 

Mr. T’s symptoms continued to worsen on IV antibiotic therapy. Dermatology was consulted, and he was diagnosed with pseudocellulitis. Gemcitabine was stopped. Pseudocellulitis treatments that have shown benefit in the literature are nonsteroidal anti-inflammatory drugs (NSAIDs), diphenhydramine, and corticosteroids in some cases (Tan et al., 2007; Korniyenko et al., 2012). Mr. T was treated with ibuprofen and topical triamcinolone 0.1% to the affected area. His symptoms improved quickly once on this regimen, and he was not given gemcitabine again.

This case is an example of a rare side effect of chemotherapy that required further investigation and a review of case reports in the literature to make a diagnosis and a treatment decision.
